# Supplementary material for: Pyruvate Dehydrogenase Kinase 1 inhibition mediated oxidative phosphorylation enhancement in cartilage promotes osteoarthritis progression
Source: BMC Musculoskelet Disord. 2023 Jul 20;24:597. doi: 10.1186/s12891-023-06585-6 (PMC10357736; doi:10.1186/s12891-023-06585-6)
Supplement: Supplementary file 1 — Additional file 1. [file 12891_2023_6585_MOESM1_ESM.zip › BP.Gsea.1653623667859/GOBP_REGULATION_OF_SIGNAL_TRANSDUCTION_BY_P53_CLASS_MEDIATOR.html]

Details for gene set GOBP\_REGULATION\_OF\_SIGNAL\_TRANSDUCTION\_BY\_P53\_CLASS\_MEDIATOR[GSEA]

|  || Dataset | 55457\_collapsed\_to\_symbols.cls.cls #OA\_versus\_normal.cls.cls #OA\_versus\_normal\_repos |
| Phenotype | cls.cls#OA\_versus\_normal\_repos |
| Upregulated in class | normal |
| GeneSet | GOBP\_REGULATION\_OF\_SIGNAL\_TRANSDUCTION\_BY\_P53\_CLASS\_MEDIATOR |
| Enrichment Score (ES) | -0.44838348 |
| Normalized Enrichment Score (NES) | -1.8995262 |
| Nominal p-value | 0.0 |
| FDR q-value | 0.057192735 |
| FWER p-Value | 0.718 |
Table: GSEA Results Summary

  

Fig 1: Enrichment plot: GOBP\_REGULATION\_OF\_SIGNAL\_TRANSDUCTION\_BY\_P53\_CLASS\_MEDIATOR      
 Profile of the Running ES Score & Positions of GeneSet Members on the Rank Ordered List

  

| SYMBOL | TITLE | RANK IN GENE LIST | RANK METRIC SCORE | RUNNING ES | CORE ENRICHMENT || 1 | PSMD10 | "proteasome 26S subunit, non-ATPase 10 [Source:HGNC Symbol;Acc:HGNC:9555]" | 58 | 0.976 | 0.0250 | No |
| 2 | ZMPSTE24 | zinc metallopeptidase STE24 [Source:HGNC Symbol;Acc:HGNC:12877] | 451 | 0.682 | 0.0150 | No |
| 3 | MIF | macrophage migration inhibitory factor [Source:HGNC Symbol;Acc:HGNC:7097] | 555 | 0.640 | 0.0263 | No |
| 4 | ING4 | inhibitor of growth family member 4 [Source:HGNC Symbol;Acc:HGNC:19423] | 680 | 0.598 | 0.0347 | No |
| 5 | RNF34 | ring finger protein 34 [Source:HGNC Symbol;Acc:HGNC:17297] | 699 | 0.594 | 0.0512 | No |
| 6 | SMYD2 | SET and MYND domain containing 2 [Source:HGNC Symbol;Acc:HGNC:20982] | 856 | 0.553 | 0.0557 | No |
| 7 | AKT1 | AKT serine/threonine kinase 1 [Source:HGNC Symbol;Acc:HGNC:391] | 1192 | 0.478 | 0.0440 | No |
| 8 | AURKB | aurora kinase B [Source:HGNC Symbol;Acc:HGNC:11390] | 1407 | 0.442 | 0.0407 | No |
| 9 | YJU2 | YJU2 splicing factor homolog [Source:HGNC Symbol;Acc:HGNC:25518] | 1832 | 0.380 | 0.0191 | No |
| 10 | PRKN | parkin RBR E3 ubiquitin protein ligase [Source:HGNC Symbol;Acc:HGNC:8607] | 1972 | 0.364 | 0.0192 | No |
| 11 | PTTG1IP | PTTG1 interacting protein [Source:HGNC Symbol;Acc:HGNC:13524] | 2012 | 0.360 | 0.0271 | No |
| 12 | TAF9B | TATA-box binding protein associated factor 9b [Source:HGNC Symbol;Acc:HGNC:17306] | 2081 | 0.352 | 0.0324 | No |
| 13 | COPS3 | COP9 signalosome subunit 3 [Source:HGNC Symbol;Acc:HGNC:2239] | 2144 | 0.345 | 0.0380 | No |
| 14 | TRIAP1 | TP53 regulated inhibitor of apoptosis 1 [Source:HGNC Symbol;Acc:HGNC:26937] | 2163 | 0.342 | 0.0469 | No |
| 15 | CHEK2 | checkpoint kinase 2 [Source:HGNC Symbol;Acc:HGNC:16627] | 2533 | 0.299 | 0.0272 | No |
| 16 | CHEK1 | checkpoint kinase 1 [Source:HGNC Symbol;Acc:HGNC:1925] | 3173 | 0.233 | -0.0156 | No |
| 17 | MLF1 | myeloid leukemia factor 1 [Source:HGNC Symbol;Acc:HGNC:7125] | 3971 | 0.158 | -0.0731 | No |
| 18 | ELL3 | elongation factor for RNA polymerase II 3 [Source:HGNC Symbol;Acc:HGNC:23113] | 4801 | 0.087 | -0.1351 | No |
| 19 | AURKA | aurora kinase A [Source:HGNC Symbol;Acc:HGNC:11393] | 4916 | 0.079 | -0.1416 | No |
| 20 | ATAD5 | ATPase family AAA domain containing 5 [Source:HGNC Symbol;Acc:HGNC:25752] | 5020 | 0.071 | -0.1475 | No |
| 21 | SPRED2 | sprouty related EVH1 domain containing 2 [Source:HGNC Symbol;Acc:HGNC:17722] | 5179 | 0.058 | -0.1581 | No |
| 22 | DYRK2 | dual specificity tyrosine phosphorylation regulated kinase 2 [Source:HGNC Symbol;Acc:HGNC:3093] | 5224 | 0.054 | -0.1599 | No |
| 23 | TP53 | tumor protein p53 [Source:HGNC Symbol;Acc:HGNC:11998] | 5511 | 0.032 | -0.1813 | No |
| 24 | BCL2 | BCL2 apoptosis regulator [Source:HGNC Symbol;Acc:HGNC:990] | 5609 | 0.023 | -0.1881 | No |
| 25 | KAT6A | lysine acetyltransferase 6A [Source:HGNC Symbol;Acc:HGNC:13013] | 5778 | 0.010 | -0.2010 | No |
| 26 | MAPKAPK5 | MAPK activated protein kinase 5 [Source:HGNC Symbol;Acc:HGNC:6889] | 6094 | -0.012 | -0.2252 | No |
| 27 | ING2 | inhibitor of growth family member 2 [Source:HGNC Symbol;Acc:HGNC:6063] | 6150 | -0.016 | -0.2290 | No |
| 28 | ATM | ATM serine/threonine kinase [Source:HGNC Symbol;Acc:HGNC:795] | 6179 | -0.019 | -0.2306 | No |
| 29 | BDKRB2 | bradykinin receptor B2 [Source:HGNC Symbol;Acc:HGNC:1030] | 6276 | -0.026 | -0.2373 | No |
| 30 | EEF1E1 | eukaryotic translation elongation factor 1 epsilon 1 [Source:HGNC Symbol;Acc:HGNC:3212] | 6296 | -0.027 | -0.2380 | No |
| 31 | TAF9 | TATA-box binding protein associated factor 9 [Source:HGNC Symbol;Acc:HGNC:11542] | 6297 | -0.027 | -0.2372 | No |
| 32 | NOP53 | NOP53 ribosome biogenesis factor [Source:HGNC Symbol;Acc:HGNC:4333] | 6334 | -0.031 | -0.2390 | No |
| 33 | MARCHF7 | membrane associated ring-CH-type finger 7 [Source:HGNC Symbol;Acc:HGNC:17393] | 6357 | -0.032 | -0.2398 | No |
| 34 | CD74 | CD74 molecule [Source:HGNC Symbol;Acc:HGNC:1697] | 6374 | -0.033 | -0.2400 | No |
| 35 | ANKRD1 | ankyrin repeat domain 1 [Source:HGNC Symbol;Acc:HGNC:15819] | 6416 | -0.037 | -0.2421 | No |
| 36 | PML | PML nuclear body scaffold [Source:HGNC Symbol;Acc:HGNC:9113] | 6421 | -0.037 | -0.2413 | No |
| 37 | CDK5RAP3 | CDK5 regulatory subunit associated protein 3 [Source:HGNC Symbol;Acc:HGNC:18673] | 6583 | -0.048 | -0.2524 | No |
| 38 | MAP2K6 | mitogen-activated protein kinase kinase 6 [Source:HGNC Symbol;Acc:HGNC:6846] | 7246 | -0.099 | -0.3011 | No |
| 39 | MDM2 | MDM2 proto-oncogene [Source:HGNC Symbol;Acc:HGNC:6973] | 7273 | -0.101 | -0.3001 | No |
| 40 | PLA2R1 | phospholipase A2 receptor 1 [Source:HGNC Symbol;Acc:HGNC:9042] | 7321 | -0.103 | -0.3006 | No |
| 41 | DYRK1A | dual specificity tyrosine phosphorylation regulated kinase 1A [Source:HGNC Symbol;Acc:HGNC:3091] | 7667 | -0.132 | -0.3236 | No |
| 42 | MUC1 | "mucin 1, cell surface associated [Source:HGNC Symbol;Acc:HGNC:7508]" | 7871 | -0.146 | -0.3350 | No |
| 43 | PRMT5 | protein arginine methyltransferase 5 [Source:HGNC Symbol;Acc:HGNC:10894] | 7904 | -0.148 | -0.3330 | No |
| 44 | HEXIM1 | HEXIM P-TEFb complex subunit 1 [Source:HGNC Symbol;Acc:HGNC:24953] | 8293 | -0.178 | -0.3579 | No |
| 45 | KDM1A | lysine demethylase 1A [Source:HGNC Symbol;Acc:HGNC:29079] | 8608 | -0.202 | -0.3764 | No |
| 46 | SIRT1 | sirtuin 1 [Source:HGNC Symbol;Acc:HGNC:14929] | 8947 | -0.227 | -0.3959 | No |
| 47 | UBB | ubiquitin B [Source:HGNC Symbol;Acc:HGNC:12463] | 9142 | -0.243 | -0.4037 | No |
| 48 | NUAK1 | NUAK family kinase 1 [Source:HGNC Symbol;Acc:HGNC:14311] | 9190 | -0.247 | -0.3999 | No |
| 49 | RPL37 | ribosomal protein L37 [Source:HGNC Symbol;Acc:HGNC:10347] | 9265 | -0.253 | -0.3980 | No |
| 50 | SOX4 | SRY-box transcription factor 4 [Source:HGNC Symbol;Acc:HGNC:11200] | 9291 | -0.255 | -0.3922 | No |
| 51 | SNAI2 | snail family transcriptional repressor 2 [Source:HGNC Symbol;Acc:HGNC:11094] | 9697 | -0.291 | -0.4151 | No |
| 52 | PLK3 | polo like kinase 3 [Source:HGNC Symbol;Acc:HGNC:2154] | 9698 | -0.291 | -0.4063 | No |
| 53 | C16orf72 | chromosome 16 open reading frame 72 [Source:HGNC Symbol;Acc:HGNC:30103] | 9902 | -0.312 | -0.4127 | No |
| 54 | TRIM24 | tripartite motif containing 24 [Source:HGNC Symbol;Acc:HGNC:11812] | 9927 | -0.314 | -0.4051 | No |
| 55 | PMAIP1 | phorbol-12-myristate-13-acetate-induced protein 1 [Source:HGNC Symbol;Acc:HGNC:9108] | 10084 | -0.329 | -0.4073 | No |
| 56 | RPL5 | ribosomal protein L5 [Source:HGNC Symbol;Acc:HGNC:10360] | 10201 | -0.341 | -0.4060 | No |
| 57 | RPS7 | ribosomal protein S7 [Source:HGNC Symbol;Acc:HGNC:10440] | 10608 | -0.384 | -0.4261 | No |
| 58 | PYHIN1 | pyrin and HIN domain family member 1 [Source:HGNC Symbol;Acc:HGNC:28894] | 10635 | -0.386 | -0.4165 | No |
| 59 | RRS1 | ribosome biogenesis regulator 1 homolog [Source:HGNC Symbol;Acc:HGNC:17083] | 11045 | -0.437 | -0.4352 | Yes |
| 60 | EIF5A | eukaryotic translation initiation factor 5A [Source:HGNC Symbol;Acc:HGNC:3300] | 11127 | -0.446 | -0.4280 | Yes |
| 61 | ATR | ATR serine/threonine kinase [Source:HGNC Symbol;Acc:HGNC:882] | 11231 | -0.461 | -0.4221 | Yes |
| 62 | RPL11 | ribosomal protein L11 [Source:HGNC Symbol;Acc:HGNC:10301] | 11252 | -0.464 | -0.4097 | Yes |
| 63 | URB2 | URB2 ribosome biogenesis homolog [Source:HGNC Symbol;Acc:HGNC:28967] | 11291 | -0.470 | -0.3984 | Yes |
| 64 | PAK1IP1 | PAK1 interacting protein 1 [Source:HGNC Symbol;Acc:HGNC:20882] | 11345 | -0.478 | -0.3881 | Yes |
| 65 | CD44 | CD44 molecule (Indian blood group) [Source:HGNC Symbol;Acc:HGNC:1681] | 11439 | -0.494 | -0.3805 | Yes |
| 66 | STK11 | serine/threonine kinase 11 [Source:HGNC Symbol;Acc:HGNC:11389] | 11451 | -0.496 | -0.3663 | Yes |
| 67 | RPS20 | ribosomal protein S20 [Source:HGNC Symbol;Acc:HGNC:10405] | 11507 | -0.502 | -0.3555 | Yes |
| 68 | RPS15 | ribosomal protein S15 [Source:HGNC Symbol;Acc:HGNC:10388] | 11671 | -0.527 | -0.3523 | Yes |
| 69 | TWIST1 | twist family bHLH transcription factor 1 [Source:HGNC Symbol;Acc:HGNC:12428] | 11696 | -0.531 | -0.3381 | Yes |
| 70 | EP300 | E1A binding protein p300 [Source:HGNC Symbol;Acc:HGNC:3373] | 11741 | -0.540 | -0.3252 | Yes |
| 71 | RRN3 | "RRN3 homolog, RNA polymerase I transcription factor [Source:HGNC Symbol;Acc:HGNC:30346]" | 11762 | -0.543 | -0.3104 | Yes |
| 72 | CHD5 | chromodomain helicase DNA binding protein 5 [Source:HGNC Symbol;Acc:HGNC:16816] | 11776 | -0.545 | -0.2949 | Yes |
| 73 | MTOR | mechanistic target of rapamycin kinase [Source:HGNC Symbol;Acc:HGNC:3942] | 12047 | -0.605 | -0.2977 | Yes |
| 74 | MSX1 | msh homeobox 1 [Source:HGNC Symbol;Acc:HGNC:7391] | 12054 | -0.609 | -0.2798 | Yes |
| 75 | HIC1 | HIC ZBTB transcriptional repressor 1 [Source:HGNC Symbol;Acc:HGNC:4909] | 12118 | -0.624 | -0.2658 | Yes |
| 76 | USP7 | ubiquitin specific peptidase 7 [Source:HGNC Symbol;Acc:HGNC:12630] | 12289 | -0.673 | -0.2587 | Yes |
| 77 | HNRNPK | heterogeneous nuclear ribonucleoprotein K [Source:HGNC Symbol;Acc:HGNC:5044] | 12341 | -0.688 | -0.2419 | Yes |
| 78 | SGK1 | serum/glucocorticoid regulated kinase 1 [Source:HGNC Symbol;Acc:HGNC:10810] | 12357 | -0.694 | -0.2221 | Yes |
| 79 | SNAI1 | snail family transcriptional repressor 1 [Source:HGNC Symbol;Acc:HGNC:11128] | 12365 | -0.696 | -0.2016 | Yes |
| 80 | NOP2 | NOP2 nucleolar protein [Source:HGNC Symbol;Acc:HGNC:7867] | 12372 | -0.699 | -0.1810 | Yes |
| 81 | DYRK3 | dual specificity tyrosine phosphorylation regulated kinase 3 [Source:HGNC Symbol;Acc:HGNC:3094] | 12379 | -0.702 | -0.1602 | Yes |
| 82 | RPL23 | ribosomal protein L23 [Source:HGNC Symbol;Acc:HGNC:10316] | 12558 | -0.777 | -0.1506 | Yes |
| 83 | BOP1 | BOP1 ribosomal biogenesis factor [Source:HGNC Symbol;Acc:HGNC:15519] | 12580 | -0.785 | -0.1286 | Yes |
| 84 | USP2 | ubiquitin specific peptidase 2 [Source:HGNC Symbol;Acc:HGNC:12618] | 12648 | -0.824 | -0.1089 | Yes |
| 85 | MYC | "MYC proto-oncogene, bHLH transcription factor [Source:HGNC Symbol;Acc:HGNC:7553]" | 12801 | -0.970 | -0.0915 | Yes |
| 86 | DDX5 | DEAD-box helicase 5 [Source:HGNC Symbol;Acc:HGNC:2746] | 12834 | -1.038 | -0.0626 | Yes |
| 87 | HIPK2 | homeodomain interacting protein kinase 2 [Source:HGNC Symbol;Acc:HGNC:14402] | 12842 | -1.045 | -0.0316 | Yes |
| 88 | TAF1 | TATA-box binding protein associated factor 1 [Source:HGNC Symbol;Acc:HGNC:11535] | 12881 | -1.192 | 0.0015 | Yes |
Table: GSEA details [plain text format]

  

Fig 2: GOBP\_REGULATION\_OF\_SIGNAL\_TRANSDUCTION\_BY\_P53\_CLASS\_MEDIATOR      
 Blue-Pink O' Gram in the Space of the Analyzed GeneSet

  

Fig 3: GOBP\_REGULATION\_OF\_SIGNAL\_TRANSDUCTION\_BY\_P53\_CLASS\_MEDIATOR: Random ES distribution      
 Gene set null distribution of ES for **GOBP\_REGULATION\_OF\_SIGNAL\_TRANSDUCTION\_BY\_P53\_CLASS\_MEDIATOR**
